# Progress in Genito-Urinary Surgery

**Published:** 1902-05-10

**Authors:** 


### Progress in Genito-Urinary Surgery.

**Cryoscopy in Genito-Urinary and Abdominal Surgery.**—A. Ogston[1] has written on this subject, and given notes of 12 cases in which the procedure gave him substantial help. Fluids freeze at a lower temperature in proportion to the quantities of impurities they contain. Korányi applied this law to the blood and urine and showed that normal blood freezes at 0·56°C. below the freezing-point of water, or at a temperature which does not vary more than one-hundredth of a degree above or below—0·56°C. The freezing-point of normal urine varies between —1°C. and—2°C. If the elimination of effete products from the blood becomes defective, the freezing-point of the blood is lowered proportionately. If, therefore, the blood freezes below—0·57°C., then elimination of waste products is defective and the patient concerned is to be regarded as a bad subject for any surgical operation, for the impurity in the blood is likely to increase for a time after an operation, and may lead to death from failure of the wound to heal, or from uræmia. These remarks apply in a special degree if any operation on the kidneys is contemplated. Ogston believes cryoscopy will be of no less value in determining the existence and degree of hepatism, a condition about which little is known, but which may threaten life and render operations dangerous. Ogston gives an illustration and description of Beckmann's apparatus which is used for the determination of the freezing-points mentioned. It consists of a jar for ice and salt into which a large test-tube dips, and within the latter is a second smaller test-tube. The blood or other fluid to be examined is placed in the smaller tube, which contains also a special thermometer. The scale on the thermometer only has a range of 6° C., each degree being divided into hundredths. The blood is drawn from any large vein about the hand, wrist, or forearm, and may be first collected in a bottle. Coagulation of the blood does not alter its freezing-point. Ogston has ceased to use cryoscopy for urine testing, believing that it gives no better indications than taking the specific gravity of the urine, but for the examination of the blood he thinks it would be difficult to exaggerate the usefulness of the method or the accuracy of the information it yields. L. Caspar (Berlin)[2] has written on the value of estimating the functional capacity of the kidneys in the diagnosis of renal or abdominal disease. The method he has used, in conjunction with P. F. Richter, consists in collecting the urine simultaneously and separately from each kidney by ureteral catheterisation, and estimating and comparing the quantities of nitrogen and of sugar (artificially produced by injecting phloridzin) in the two samples, and also in comparing their freezing-points. The three estimates in both samples will be equal if both kidneys are healthy, but lower in the secretion from the diseased side in unilateral disease, and that in a degree proportionate to the extent of the disease. The method has yielded valuable assistance in the diagnosis of obscure abdominal cases. Several illustrative cases are given by the writer. In one case the method enabled him to determine which kidney contained a calculus, though all other methods failed to indicate which side was affected. In another case the method proved that a tumour in the renal region was not a true renal tumour. An operation later showed this tumour to be an adenoma of the supra-renal capsule. In a third case essential integrity of the kidney substance was proved to exist, though an enlargement from cysts was present. In a fourth case by the method a differential diagnosis was effected between renal tumour and a perityphlitic abscess in the renal region, and in a fifth between biliary and renal colic. And finally, in three cases in which nephralgia gave symptoms almost exactly like those of stone in the kidney, the method enabled the surgeon to reject the latter diagnosis.

**Renal Calculus.**—J. Hutchinson, jun.,[3] has discussed the operative treatment of this disease. Having regard to the structure of the kidney, its nervous arrangements, and abundant vascular supply, he considers that the guiding principle should be to inflict the least possible injury in extracting a stone from the organ. In many cases, for example, it is neither advisable, easy, nor safe to split the organ open from its convex surface, nor to bring it out into the wound for that or other purposes. Such methods may rupture the renal pedicle, or cause serious hæmorrhage from the incision, or produce a urinary fistula. The pelvis of the kidney and the ureter can be better explored through a pelvic incision than through a cortical one, and the idea that the former incisions do not heal so readily as the latter is largely erroneous. It is alleged that the skilled use of the Roentgen rays will now rarely fail to demonstrate the exact position and size of any renal stone. Thus operation can be performed before the kidney has become extensively degenerated, and in doubtful cases the absence of stone may be proved and the patient saved an unnecessary operation. Under the guidance of the X-rays the operation becomes merely that for the extraction of a somewhat deeply-placed foreign body. There will be nothing gained by making large incisions, dragging the kidney into the light of day, and prodding it with needles. From personal experience Hutchinson is able to state that small clean wounds of the renal pelvis or ureter heal soundly and quickly if no roughness is used in extracting the stone and if the urine be kept aseptic with urotropine or salol. No renal calculi are so small that they can be neglected, and the X-rays will enable an early operation to be performed. It is almost an axiom in renal and vesical cases that the smaller the stone the greater the suffering produced and *vice versâ*. Two cases are contrasted, one in which a stone had been forming for 16 years, causing intense suffering and yet hardly injuring the kidney; the other, a case of Morris's, in which the stone had been forming also for 16 years, but without pain, and with the final production of a large peri-nephritic swelling, and a fatal ending after nephrectomy. If a stone is present the chances are very strong that it will be in the pelvis of the kidney, or in the upper end of the ureter, opposite the ilio-costal interval, and these are favourable positions for exploration with the X-rays. If the stone is in the pelvic portion of the ureter the photograph must be taken obliquely to avoid the shadow of the sacrum and other pelvic bones. In some cases of renal stone the pain is on one side and the stone on the other, and in such cases the X-rays will prevent operation being performed on the wrong side. In very fat subjects the rays will be valueless. An exposure of 15 seconds usually suffices to show the stone, and for that length of time the patient can hold his breath and thus prevent the kidney moving. The writer quotes Leonard's paper, giving the results of the examination of 59 suspected cases of renal calculus. The results were negative in 47, and in only one of these was it subsequently proved that a calculus had been overlooked, and that was explained by too small a plate having been used. Positive results were yielded in 12 cases, all of which were confirmed later by operation. The writer then considers the question whether an operation for so-called nephralgia is justifiable when skiagraphy gives negative results. The operation is not without risk, and any relief obtained is usually not permanent, and in some cases the patient is worse after the nephrotomy. Causes of nephralgia other than stone are pyelitis or nephritis, often localised; commencing tubercular disease; secretion of uric acid or oxalate crystals; biliary calculi, and appendicitis. In none of these would nephrotomy be of use.

**Encysted Vesical Calculus.**—T. R. Jessop[4] describes three cases in which he performed supra-pubic cystotomy for the above condition. The first case was that of a young man who had had severe symptoms of stone for a long period. A lithotrite passed into the bladder grazed the under surface of the stone, but all attempts to seize it proved futile. Bimanually with the left forefinger in the rectum and the right hand on the abdomen the calculus could be felt fixed beneath the pubic arch. Cystotomy revealed the stone to be embedded to about three-fourths of its extent in a sacculus occupying a space below the pubic arch and between it and the prostatic urethra. Recovery was uneventful. The second case was that of a man of 73. A sound could not be passed easily owing to the existence of a swelling at the base of the bladder, which was supposed to be an enormously enlarged prostate. Cystotomy showed the swelling was due to a calculus weighing 13 drachms, occupying a pouch on the right side of the base of the bladder. Only a small part of the stone was exposed at the neck of the sacculus. Recovery followed removal of the stone. In the third case also the stone occupied a pouch at the base of the bladder, and by the rectum was felt projecting towards the sacrum on the left side. Jessop remarks incidentally that he believes perineal lithotomy is doomed ere long to become a subject for the historian alone.

**Vesical Sacculus, a Cause of Intestinal Obstruction.**—Thomas Bryant[5] reports a case of this kind. The patient was a man of 67, who had had intestinal obstruction for five days, and fœtid vomiting for 12 hours. For nearly two years he had had a tumour in the right lower abdomen. The abdomen was distended chiefly in the left lower part. In the right lower part, extending up to the umbilicus, was a resistant, dull, sausage-like swelling. The urine was normal. Laparotomy was performed, and the intestinal obstruction found to be due to the pressure of the sausage-shaped swelling. This was attached to the bladder. Catheterisation did not empty it, but with pressure it was emptied into the bladder, the contents being like clear urine. The communication with the bladder was a small one. The patient died 22 hours later, though the obstruction was relieved. Reginald Harrison mentions two cases in some respects similar to the above. In one, with a large posterior vesical sacculus, there were long bouts of obstinate constipation from pressure on the rectum. This trouble was removed by draining the pouch. In the other a large suppurating sacculus was found adherent to and constricting a coil of small intestine.

1 Lancet, Nov. 9, 1901, p. 1253. 2 Centralb. f. Chir., Nov. 2, 1901, p. 1073. 3 B. M. J., Oct. 19, 1901, p. 1130. 4 Lancet, Dec. 14, 1901, p. 1653. 5 B. M. J., Nov. 30, 1901, p. 1599.

